# Meta-Analysis on the Efficacy and Safety of Hyperbaric Oxygen as Adjunctive Therapy for Vascular Dementia

**DOI:** 10.3389/fnagi.2019.00086

**Published:** 2019-04-17

**Authors:** Qiang You, Lan Li, Su-qin Xiong, Yu-fen Yan, Dan Li, Na-na Yan, Hong-ping Chen, You-ping Liu

**Affiliations:** ^1^Standardization Education Ministry Key Laboratory of Traditional Chinese Medicine, Department of Pharmacy, Chengdu University of Traditional Chinese Medicine, Chengdu, China; ^2^The Affiliated Hospital, Southwest Medical University, Luzhou, China; ^3^Department of Nursing, Southwest Medical University, Luzhou, China

**Keywords:** hyperbaric oxygen therapy, complementary therapy, systematic review, meta-analysis, vascular dementia

## Abstract

**Background:** Vascular dementia (VD) is a common type of disease in the elderly. Numerous clinical trials have suggested that hyperbaric oxygen is an effective and safe complementary therapy for aging-related disorders. However, there is no reliable systematic evidence regarding hyperbaric oxygen therapy (HBOT) for the treatment of VD. Therefore, we performed a meta-analysis to evaluate the clinical efficacy and safety of HBOT in treating VD.

**Methods:** We methodically retrieved the clinical studies from eight databases (PubMed, Cochrane Library, Embase, Web of Science, Sino-Med, China National Knowledge Infrastructure (CNKI), China Science and Technology Journal Database (VIP), and WanFang) from their inception to November 2018. RevMan 5.3.5 was used for quality assessment and data analysis. Stata 15.1 was employed for publication bias detection and sensitivity analysis.

**Results:** Twenty-five randomized clinical trials (RCTs) involving 1,954 patients met our inclusion criteria. These articles researched the HBOT + oxiracetam + conventional therapy (CT) vs. oxiracetam + CT (*n* = 13), HBOT + butylphthalide +CT vs. butylphthalide + CT (*n* = 5), HBOT + donepezil + CT vs. donepezil + CT (*n* = 4), HBOT + nicergoline + CT vs. nicergoline + CT (*n* = 2) and HBOT + CT vs. CT (*n* = 1). The results indicated that additional HBOT strikingly improved the Mini-Mental State Examination (MMSE) (*MD* = 4.00; 95% *CI* = 3.28–4.73; *P* < 0.00001), activities of daily living (ADL) (*MD* = −5.91; 95% *CI* = −6.45, −5.36; *P* < 0.00001) and ADL by Barthel index (BADL) (*MD* = 13.86; 95% *CI* = 5.63–22.10; *P* = 0.001) and increased the total efficacy rate (TEF) (*OR* = 4.84, 95% *CI* = 3.19–7.33, *P* < 0.00001). The adverse events rates were not statistically significant between the HBOT and CT groups (*OR* = 0.85, 95% *CI* = 0.26–2.78, *P* = 0.79).

**Conclusion:** In view of the effectiveness and safety of HBOT, the present meta-analysis suggested that HBOT can be recommended as an effective and safe complementary therapy for the treatment of VD.

**Protocol Registration:** PROSPERO (ID: CRD42019117178). Available online at: http://www.crd.york.ac.uk/PROSPERO/display_record.asp?ID=CRD42019117178.

## Introduction

Vascular dementia (VD), with clinical manifestations of cognitive disorder, cerebrovascular pathologies and progressive memory decline (O'Brien and Thomas, 2015), leads to more than 20% of all aphronesia cases worldwide and is second only to Alzheimer's disease (AD) (Gorelick et al., 2011). In particular, VD mainly affects patients in developing countries due to poorer health care and control of cardiovascular risk factors. It has been reported that in certain Asian countries, the prevalence of VD in 65-year-olds is 0.6–2.1% (Kalaria et al., 2008). Considering the rapid aging of the global population, especially in Asia (Ferri et al., 2005), and the increasing incidence rate of cardiovascular disease, there will be a tremendous increase in the morbidity of VD among the elderly within the next few decades (Barker et al., 2014; Etherton-Beer, 2014).

The main risk factors include hypertension, obesity, diabetes, hyperlipidemia, metabolic syndromes, dyslipidemia, cardiac diseases, smoking, hyperhomocysteinemia and genetic disposition (Levine and Langa, 2011; Sahathevan et al., 2012; Yates et al., 2012; Hasnain and Vieweg, 2014). Transient ischemic attack caused by stroke or acute cerebral infarction, which does enormous damage to cerebral vessels, is regarded as the major pathogenic factor of VD (Kalaria et al., 2016), and cerebrovascular diseases (CVDs) leading to lower cognitive performance for both VD and AD, and this has been widely accepted (O'Brien and Markus, 2014). VD patients not only endure lower quality of life, psychological and physical harm but also pose significant medical and financial burdens on families and society. Thus, the research into effective treatment of VD is of great social and clinical significance.

Currently, conventional therapy (CT), including anticoagulant drugs, dilating cerebral vessels, reducing blood viscosity, correcting electrolyte disorders and controlling cerebral edema, has been mostly concentrated on symptomatic management and reduction of underlying risk factors for cerebrovascular disease (Sorrentino et al., 2008). The cholinesterase inhibitors (donepezil, rivastigmine, and galantamine) and non-cholinergics (memantine, nimodipine, and hydergine) have been considered effective drugs for VD (Chen et al., 2011; Jellinger, 2014). However, because of the frequent contraindications, side effects and the unclear mechanism of its pathology, the effect of CT remained limited. Recently, complementary therapy that can substantially improve the disease has been proposed.

Hyperbaric oxygen therapy (HBOT) as an adjuvant treatment with the curative administration of 100 % oxygen in an elevated pressure environment of more than 1.4 atmosphere absolute (García-Covarrubias and Cuauhtémoc Sánchez-Rodríguez, 2000) has shown therapeutic effects in the treatment of VD. A typical treatment consists of 100% oxygen at 0.2 MPa for 60–120 min (Sanchez, 2013). According to the result of previous studies, the possible mechanism of HBOT in treating VD mainly comprises: increasing oxygen supply, raising the oxygen partial pressure of the tissue, decreasing intracranial pressure, relieving brain edema, promoting tissue healing and angiogenesis, improving metabolism, reducing apoptosis, alleviating oxidative stress, increasing mitochondrial function and promoting cell differentiation and regeneration (Robertson and Hart, 1999; Feldmeier and Undersea and Hyperbaric Medical Society, 2003; Wang et al., 2016). Though not fully understood, the efficacy and safety of HBOT was indisputable. A 2012 Cochrane review performed by Xiao et al tried to assess the effectiveness and safety of HBOT for VD. However, they failed to provide sufficient and reliable clinical evidence for HBOT in treating VD because only one randomized controlled trial involving 64 patients was included in their system review (Xiao et al., 2012). Therefore, it is necessary to conduct a meta-analysis again by collecting more clinical data to evaluate the efficacy and safety of HBOT for VD.

## Methods

### Search Strategy

The Cochrane library, Web of science, PubMed, Embase, CNKI (China National Knowledge Infrastructure), Wan-Fang, VIP (Chinese Science and Technology Periodical Database), and Sino-Med (Chinese Biomedical) were systematically searched from inception to November 2018. To obtain the maximum possible number of RCTs, we searched the above four commonly used Chinese databases. In brief, we used the following search strategies: subject terms + entry terms: (1) [Title/abstract] (“Hyperbaric oxygen” *OR* “Hyperbaric Oxygenation” *OR* “HBOT” *OR* “Oxygen Therapy” *OR* “High pressure oxygen” *OR* “HPO”); (2) Title/abstract: (“dementia^*^” *OR* “aphronesia^*^” *OR* “Amentia^*^” *OR* “VD” *OR* “VaD”). Then, (1) and (2) were connected with “AND.” In terms of the Chinese databases, we used the following key words: Gaoyayang [Title/Abstract] and Chidai [Title]. The search results were imported to document management software for the further screening.

### Study Selection and Data Extraction

Inclusion criteria: (1) if the study was an RCT performed in humans, whether they were blinded or not. (2) Patients were diagnosed with VD according to “Diagnostic and statistical manual of mental disorders DSM-IV (Christopher, 1994),” issued by American Psychiatric Association (APA), or “Draft diagnostic criteria for VD (DDC-VD)” (Qian et al., 2002) published by Neurological branch of Chinese Medical Association, or other accepted diagnostic criteria for VD. (3) The experimental group was treated with HBOT and conventional therapy (CT), and the control group was treated with conventional therapy without regard to the treatment duration, age, course of disease, sex, and ethnicity. (4) At least one or more outcome indicator, including MMSE, ADL, BADL and TEF, was applied to evaluate the curative effect. Exclusion criteria: (1) Non-RCTs, (2) animal experiments, (3) systematic review and case reports, (4) incorrect or incomplete data, and (5) conventional treatment was inconsistent between the control group and the experimental group. Two reviewers (Nana Yan and Dan Li) independently extracted the data of the literature, including the following contents: general trial characteristics (first author's last name, publication date, study period and diagnostic criteria); baseline patient and disease data (number of patients in each group and age); interventions (HBOT name, Western medicine name, treatment duration and dose), outcome definitions, and detailed adverse reactions. Discrepancies were resolved by consensus or a third researcher.

### Quality Assessment

Two reviewers (Yufen Yan and Suqin Xiong) independently assessed the methodological qualities of the trials in accordance with the Cochrane manual delineated in version 5.3.5. The risk of bias consisted of seven items: selection bias, performance bias, detection bias, attrition bias, reporting bias, and other bias. Each item was classified into low bias risk, high bias risk, and unclear bias risk. Disagreements between reviewers were settled through discussion.

### Statistical Analysis

In this review, the statistical analyses were conducted by reviewer manager (version 5.3.5), and *OR* presented with 95% confidence intervals (*CI*) (employed for the analyses of dichotomous data), whereas the continuous data were presented as *MD* with 95% *CI*. A fixed-effects and random- effects model was used to merge the data according to heterogeneity, which was determined using the chi-square test. With the *I*^2^ statistic, an *I*^2^ < 25%, indicates that heterogeneity may not be important, a value between 25 and 50% represents moderate inconsistency, and *I*^2^ > 50% suggest severe heterogeneity. We defined *P* ≥ 0.1 and *I*^2^ < 50 as an indicator that the results have good agreement and that the fixed-effects model (FEM) may be set, while *I*^2^ > 50% was defined as an indicator of striking heterogeneity between the data. Then, a random-effects model was employed to pool the results to minimize the influence of potential clinical heterogeneity. Stata 15.1 was used for detection of the possible sources of significant heterogeneity by sensitivity analysis. Publication bias was detected by Egger's test. *P* < 0.05 suggested that there was publication bias. Subgroup analysis was conducted to assess differential associations between studies based on: (1) HBOT + CT + oxiracetam vs. CT + oxiracetam, (2) HBOT + CT + butylphthalide vs. CT + butylphthalide, (3) HBOT + CT + donepezil vs. CT + donepezil, (4) HBOT + CT + nicergoline vs. CT + nicergoline, and (5) HBOT + CT vs. CT.

## Results

### Study Inclusion and Characteristics

**Figure 1:**
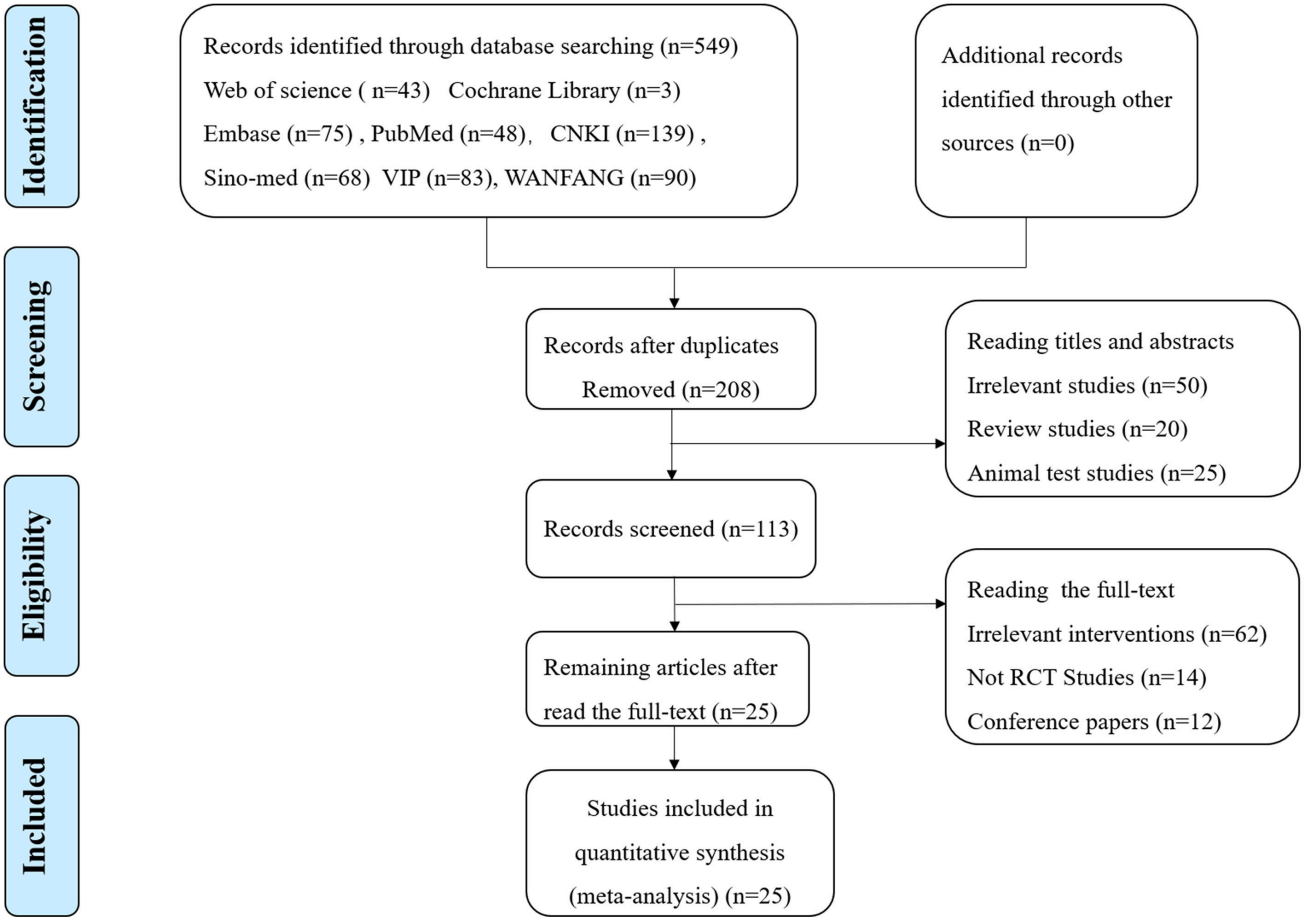
The flowchart of study selection.

Of the 549 potentially relevant studies searched from the eight databases, 341 duplicated publications were removed, and 208 papers were left for further screening. After reading titles and abstracts, there were 113 reports left, and 87 articles were excluded for one of following reasons: (1) irrelevant study, (2) not a randomized controlled trial, (3) conference papers. Finally, the remaining 25 RCTs with a total of 1.954 patients meeting our inclusion criteria (983 for HBOT group and 971 for control group) were included in the final review (Figure 1). Additionally, all trials were performed and published in China. Among the 25 studies, five comparisons were employed between the HBOT group and the control group, including HBOT+CT+ oxiracetam vs. CT+ oxiracetam (13 RCTs), HBOT+CT+ butylphthalide vs. CT+ butylphthalide (5 RCTs), HBOT+ CT+ donepezil vs. CT+ donepezil (4 RCTs), HBOT+ CT+ nicergoline vs. CT +nicergoline (2 RCTs), and HBOT+CT vs. CT (1 RCT). The treatment duration lasted from 3 to 16 weeks, and sample sizes varied from 40 to 156. Twelve studies reported specific adverse events (Table 1).

**Table 1:** The baseline characteristics of the 25 studies.

**Study**	**Study period/diagnostic criteria**	**Sample size**		**Age**		**Intervention**		**Treatment duration**	**Main outcome**	**Adverse events**
		**E (M/F)**	**C (M/F)**	**E**	**C**	**E**	**C**			
Liu, 2016	2015–2016/DSM-IV	32 (19/13)	32 (18/14)	62.8 ± 7.1	62.2 ± 7.5	HBO (60 min, qd, 0.2–0.25 MPa) CT+ Oxiracetam (0.8 g, tid, PO)	CT+ Oxiracetam (0.8g, tid, PO)	7 weeks	MMSE, ADL	NR
Qiao, 2016	2013–2015/DSM-IV	78 (42/36)	78 (45/33)	66 ± 11	65 ± 11	HBO (60 min, qd, 0.2–0.25 MPa) CT+ Oxiracetam (4 g, qd, ivgtt)	CT+ Oxiracetam (4 g, qd, ivgtt)	4 weeks	TEF, MMSE, ADL, Hemorheology	NR
Lei, 2016	2014–2016/DDC-VD	30 (16/14)	30 (17/13)	66.8 ± 3.7	66.7 ± 3.9	HBO (60 min, qd, –) CT+ Oxiracetam (0.8 g, tid, PO)	CT+ Oxiracetam (0.8 g, tid, PO)	3 weeks	TEF, MMSE, ADL	No
Xu, 2015	2013–2015/DSM-IV	50 (30/20)	50 (28/22)	56.7 ± 1.01	58.75 ± 2.01	HBO (60 min, qd, 0.2–0.25 MPa) CT+ Oxiracetam (0.8 g, tid, PO)	CT+ Oxiracetam (0.8 g, tid, PO)	7 weeks	TEF, MMSE, ADL	NR
Xia, 2012	2008–2010/DSM-IV	30 (16/14)	30 (13/17)	55–73	58–76	HBO (60 min, qd, 0.15–0.2 MPa) CT+ Oxiracetam (0.8 g, tid, PO)	CT+ Oxiracetam (0.8 g, tid, PO)	4 weeks	MMSE, ADL	E: 2 cases: Anxiety, Sleep disorders C: 2 case: Dizziness, insomnia
Wang and Zhai, 2011	2010–2011/DSM-IV	40 (22/18)	40 (20/20)	64.2 ± 7.2	63.7 ± 9.2	HBO (60 min, qd, 0.2–0.25 MPa) CT+ Oxiracetam (0.8 g, tid, PO)	CT+ Oxiracetam (0.8 g, tid, PO)	7 weeks	MMSE, ADL	NR
Wu et al., 2010	2007–2009/DSM-IV	50 (27/23)	50 (28/22)	64.2 ± 1.90	63.2 ± 2.11	HBO (60 min, qd, –) CT+ Oxiracetam (4 g, qd, ivgtt)	CT+ Oxiracetam (4 g, qd, ivgtt)	3 weeks	TEF, MMSE	E: 1 case: elevated blood pressure Increased Heart Rate, Rhinorrhagia
Chen, 2011	2008–2010/DSM-IV	41 (22/19)	41 (21/20)	64.2 ± 7.2	63.7 ± 9.1	HBO (60 min, qd, 0.2–0.25 MPa) CT+ Oxiracetam (0.8 g, tid, PO)	CT+ Oxiracetam (0.8 g, tid, PO)	7 weeks	MMSE, ADL	NR
Bu, 2012	2011–2012/DSM-IV	32 (–/–)	32 (–/–)	>40	>40	HBO (60 min, qd, –) CT+ Oxiracetam (4 g, qd, ivgtt)	CT+ Oxiracetam (4 g, qd, ivgtt)	3 weeks	TEF, MMSE	No
Li, 2013	2009–2012/DSM-IV	36 (21/15)	32 (20/16)	65.2 ± 6.9	65.3 ± 6.9	HBO (60 min, qd, 0.2–0.25 MPa) CT+ Oxiracetam (0.8 g, tid, PO)	CT+ Oxiracetam (0.8 g, tid, PO)	7 weeks	MMSE, ADL	NR
Jian, 2013	2011–2013/DSM-IV	30 (22/8)	30 (20/10)	65.1 ± 9.5	63 ± 8.6	HBO (60 min, qd, 0.2–0.25 MPa) CT+ Oxiracetam (4 g, qd, ivgtt)	CT+ Oxiracetam (4 g, qd, ivgtt)	3 weeks	MMSE, ADL	E: 3 cases: Epigastric discomfort, nausea, Digestive Symptoms. C: 2 cases: Headache, dizziness, drowsiness
Zhou, 2017	2014–2015 –	35	35	42–80	42–80	HBO (60 min, qd, 0.2 MPa) CT+ Oxiracetam (4 g, qd, ivgtt)	CT+ Oxiracetam (4 g, qd, ivgtt)	3 weeks	TEF, MMSE, BADL	NR
Yu, 2016	2014–2015/DSM-IV	38 (22/16)	42 (25/17)	64.7 ± 8.3	65.7 + 9.3	HBO (60 min, qd, 0.2 MPa) CT+ Oxiracetam (0.8 g, tid, PO)	CT+ Oxiracetam (0.8 g, tid, PO)	7 weeks	MMSE, BADL	NR
Feng, 2015	2011–2014/DDC-VD	45 (26/19)	44 (26/18)	61.4 ± 6.5	61.3 ± 6.6	HBO (60 min, qd, 0.2 MPa) CT+ Butylphthalide (0.2 g, tid, PO)	CT+ Butylphthalide (0.2 g, tid, PO)	12 weeks	MMSE, BADL, CDR	NR
Sun et al., 2015	2013–2014/ –	30 (16/14)	30 (18/12)	67.0 ± 4.9	68.0 ± 5.6	HBO (60 min, qd, 0.2 MPa) CT+ Butylphthalide (0.2 g, qid, PO)	CT+ Butylphthalide (0.2 g, qid,PO)	3 weeks	TEF, MMSE	E: 2 cases: Nausea, abdominal pain C: 9 cases: Nausea, abdominal pain, Mental abnormalities
Zhao, 2015	2010–2013/DDC-VD	83 (–/–)	83 (–/–)	65–75	65–75	HBO (60 min, qd, 0.2 MPa) Butylphthalide (0.2 g, tid, PO)	CT+ Butylphthalide (0.2 g, tid, PO)	12 weeks	MMSE, BADL,	10 Cases: Nausea, vomiting, dizziness, headache, elevated ALT
Xue, 2017	2015–2016/ –	40 (16/24)	40 (18/22)	67 ± 4.9	68 ± 5.6	HBO (60 min, qd, 0.2 MPa) CT+ Butylphthalide (0.2 g, qid, PO)	CT+ Butylphthalide (0.2 g, qid, PO)	3 weeks	TEF	E: 2 cases: Nausea, abdominal pain C: 11 cases: Nausea, abdominal pain Mental abnormalities
Wu and Tang, 2016	2013–2015/DSM-IV	30 (19/11)	30 (20/10)	68.85 ± 17.95	66.18 ± 18.52	HBO (60 min, qd, –) Butylphthalide (0.2 g, tid, PO)	CT+ Butylphthalide (0.2 g, tid, PO)	3 weeks	TEF, MMSE	E: 1 case: Tinnitus, Palpitations
Wu and Xu, 2008	–/ DDC-VD	20 (11/9)	20 (8/20)	65.2 ± 6.1	66.4 ± 5.8	HBO (60 min, qd, 0.2 MPa) CT+ Donepezil (5 mg, qd, PO)	CT+ Donepezil (5 mg, qd, PO)	12 weeks	MMSE, ADL	E: Earache in 4 case, Mild diarrhea in 1 case C: epigastric pain in 1 case
Yuan and Shi, 2010	2004–2008/DSM-IV	27 (15/12)	21 (12/9)	65.1 ± 7.1	64.4 ± 7.9	HBO (120 min, qd, 0.15–0.2 MPa) CT+ Donepezil (5 mg, qd, PO)	CT+ Donepezil (5 mg, qd, PO)	12 weeks	TEF, MMSE, ADL, WMS	11 Cases: Nausea, gastrointestinal, discomfort, 6 Cases: Insomnia
Wang et al., 2009	2005–2007/DSM-IV	32 (20/12)	32 (21/11)	70.4 ± 8.5	70.8 ± 8.1	HBO (60 min, qd, 0.2 MPa) CT+ Donepezil (5 mg, qd, PO)	CT+ Donepezil (5 mg, qd, PO)	12 weeks	MMSE, HDS	NR
Jing and Luo, 2009	–/ DSM-IV	32 (20/12)	33 (19/14)	66.12 ± 6.78	66.18 ± 7.16	HBO (120 min, qd, –) CT+ Donepezil (5 mg, qd, PO)	CT+ Donepezil (5 mg, qd, PO)	3 weeks	MMSE, MBI, BDAE	5 cases: Dizziness, Nausea, Diarrhea
Wei, 2014	2010–2012/DSM-IV	30	30	–	–	HBO (60 min, qd, 0.2MPa) CT+ Nicergoline (10–20 mg, bid, PO)	CT+ Nicergoline (10–20 mg, bid, PO)	16 weeks	MMSE, BADL	NR
Wang, 2017	2016–2017/ –	56 (38/18)	46 (3016)	66.14 ± 3.05	65.28 ± 2.41	HBO (60 min, qd, 0.2 MPa) CT+ Nicergoline (10–20 mg, bid, PO)	CT+ Nicergoline (10–20 mg, bid, PO)	8 weeks	MMSE, ADL	NR
Deng et al., 2011	2009–2010/DSM-IV	36 (20/16)	36 (22/14)	65.1 ± 6.8	63.5 ± 7.2	HBO (60 min, qd, 0.2 MPa) CT	CT	12 weeks	MMSE, BADL, Hemorheology	NR

*E, experiment group; C, control group; HBO, hyperbaric oxygen; PO, per os; ID, intravenous drip; Ivgtt, intravenous guttae; TEF, Total efficacy rate; MMSE, mini-mental state examination; ADL, activities of daily living; BADL, ADL by Barthel index; NR, no record; BDAE, Boston diagnostic aphasia test; HDS, Hasegawa dementia scale; WMS, Wechsler memory scale; CDR, clinical dementia rating; MBI, modified Barthel index. DSM-IV, “Diagnostic and statistical manual of mental disorders,” issued by American Psychiatric Association (APA); DDC-VD, “Draft diagnostic criteria for VD,” issued by neurological branch of Chinese Medical Association*.

### Methodological Quality Assessment

**Figure 2:**
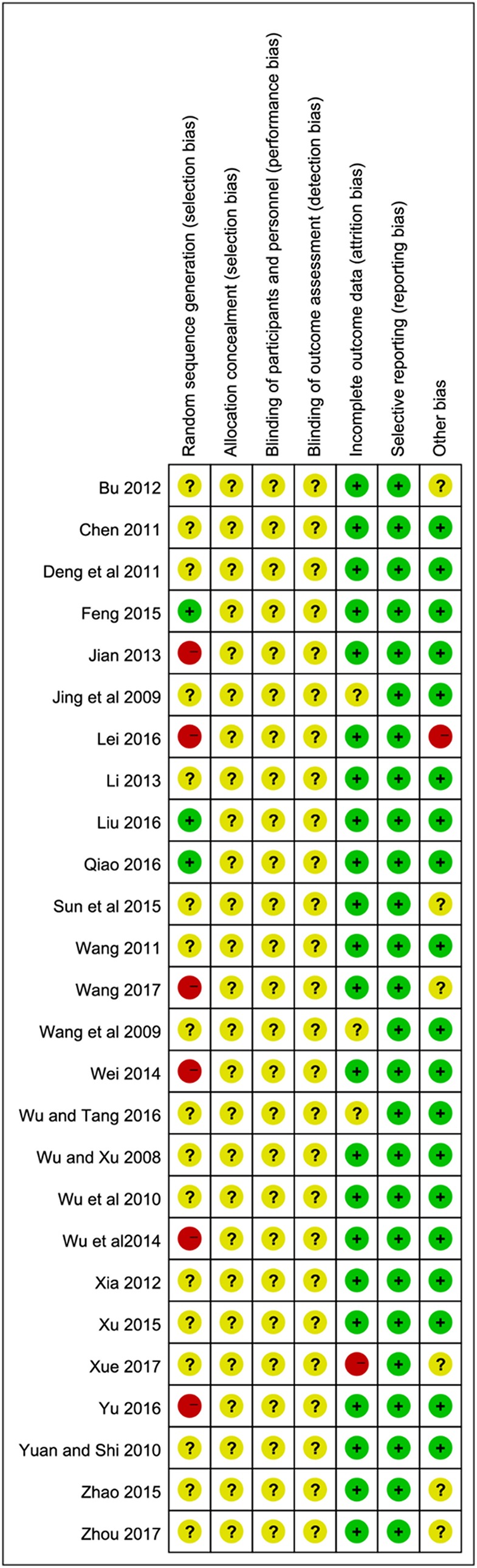
Risk of bias assessment of the 25 trials.

Among the 25 included trials, three studies (Feng, 2015; Liu, 2016; Qiao, 2016) applied random number tables for random sequence generation. Therefore, we considered them to be low risk. Five trials (Jian, 2013; Wei, 2014; Lei, 2016; Yu, 2016; Wang, 2017) were high risk due to using non-standard randomized methods, including registration order, odd-even sequence of the case caudal numbers or therapeutic regimen, while the remaining 17 studies did not offer any detailed information regarding the generation of random sequence. Almost all the studies failed to give the specific allocation concealment, performance bias and detection bias. In terms of incomplete outcome data, one study (Xue, 2017) was at high risk for the absence of a detailed MMSE score. Overall, the quality of the article is relatively low or remained indistinct because the unclear risk of biases took up a large proportion in their research. The particular results of bias assessment are summarized in Figure 2.

### Primary Outcomes

#### TEF: Addition of HBOT vs. Conventional Therapy

**Figure 3:**
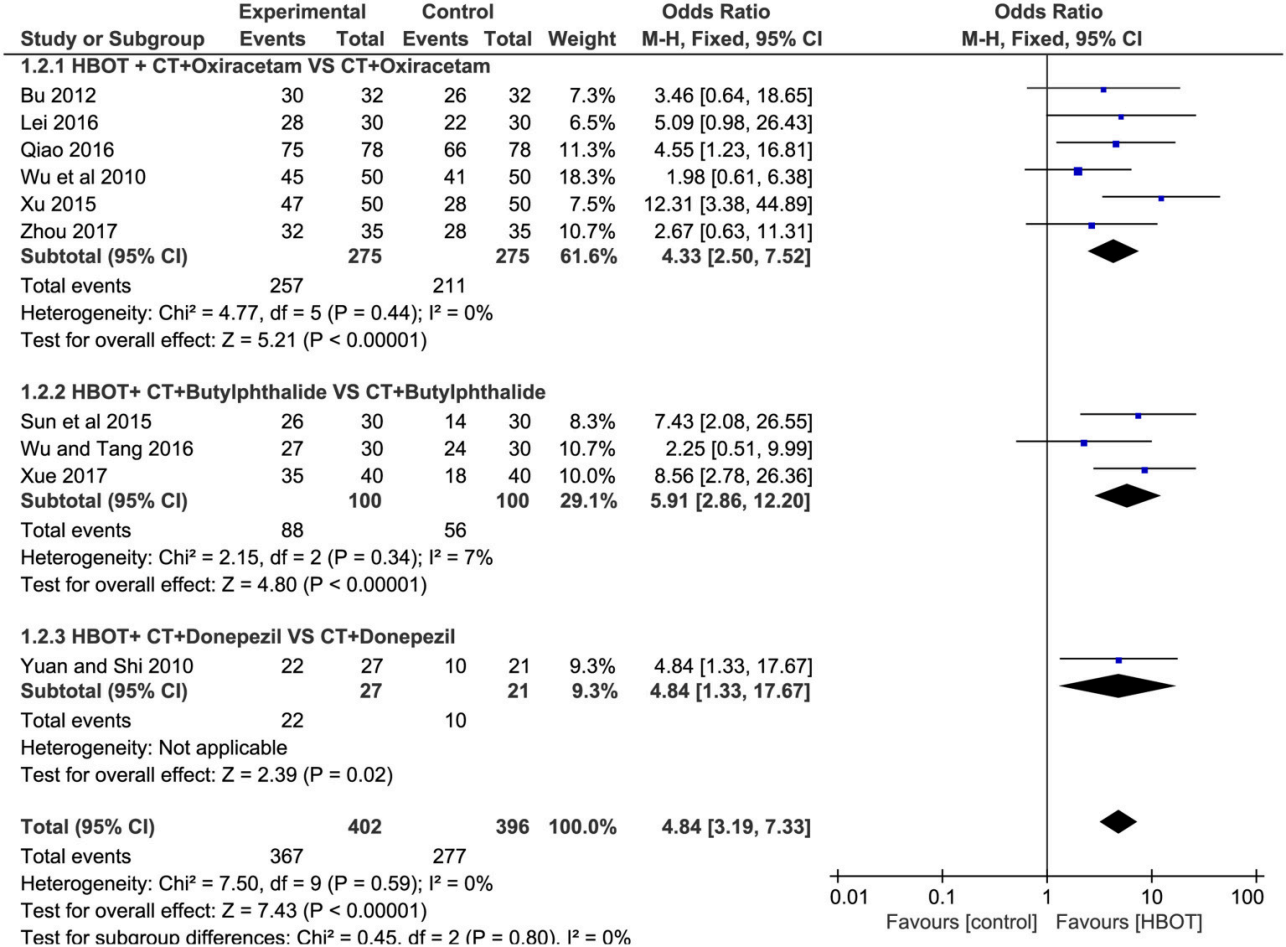
Subgroup analysis of the TEF: (1) HBOT+CT+ oxiracetam vs. CT+ oxiracetam, (2) HBOT+CT+ butylphthalide vs. CT+ butylphthalide, and (3) HBOT+CT+ donepezil vs. CT+ donepezil.

Ten studies involving 798 (40.8%) participants reported the TEF based on the MMSE score, with 402 (40.9%) patients randomized to receive additional HBOT and 396 (40.8%) patients randomized to receive CT. No heterogeneity was observed after the heterogeneity test (*P* = 0.59, χ^2^ = 0.42, *I*^2^ = 0%), and the fixed-effects model was selected for merging the data. The results showed a statistically significant difference between the HBOT and control groups, which suggested that the treatment of VD with addition of HBOT was better than routine treatment in terms of the TEF (OR = 4.84, 95% CI = 3.19–7.33, *P* < 0.00001), and no differences existed by subgroup analysis (*P* = 0.8, *I*^2^ = 0%), as shown in Figure 3.

#### MMSE Score: Addition of HBOT vs. Conventional Therapy

**Figure 4:**
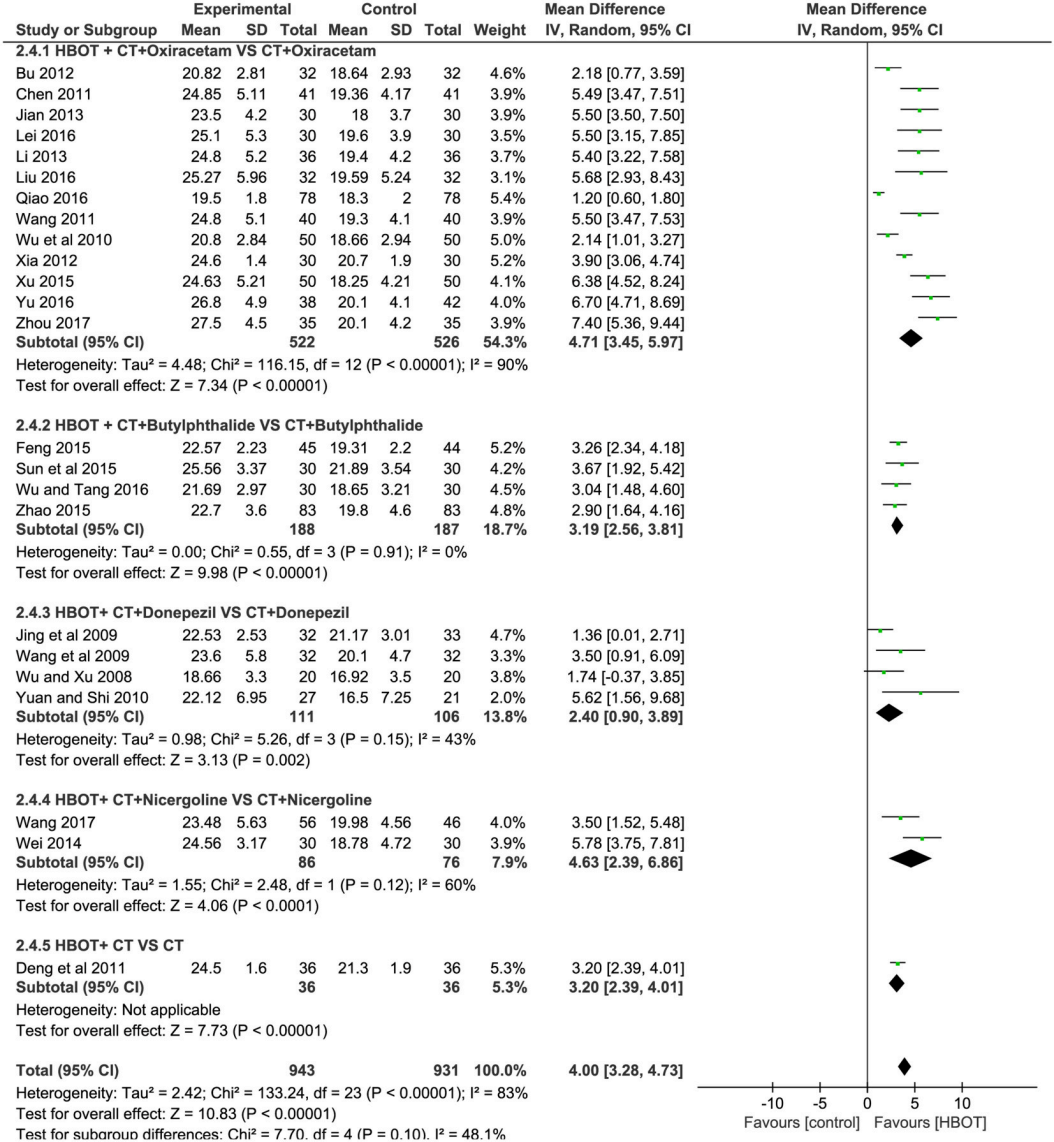
Subgroup analysis of the MMSE score after treatment (2.4.1) HBOT+CT+ oxiracetam vs. CT+ oxiracetam, (2.4.2) HBOT+CT+ butylphthalide vs. CT + butylphthalide, and (2.4.3) HBOT+CT+ donepezil vs. CT+ donepezil, (2.4.4) HBOT+CT+ nicergoline vs. CT+ nicergoline, (2.4.5) HBOT+CT vs. CT.

Of the included articles, all the studies except one (Xue, 2017) employed the MMSE to evaluate the addition of HBOT in the improvement of curative effects. Considering the significant heterogeneity (*P* < 0.00001, *I*^2^ = 83%), we synthesized the data using a random-effects modal and introduced subgroup analysis including (1) HBOT + oxiracetam+ CT vs. oxiracetam + CT (*n* = 13), HBOT + butylphthalide + CT vs. butylphthalide + CT (*n* = 4), HBOT + donepezil + CT vs. donepezil + CT (*n* = 4), HBOT + nicergoline + CT vs. nicergoline + CT (*n* = 2) and HBOT + CT vs. CT (*n* = 1). The pooled results strikingly favored the HBOT +CT group (*MD* = 4.00; 95% *CI* = 3.28–4.73; *P* < 0.00001). Subgroup analysis showed that no statistically significant difference was observed among the five groups (*P* = 0.1, *I*^2^ = 48%), and the heterogeneity mainly comes from the group treated with oxiracetam (*MD* = 4.71; 95% *CI* = 3.45–5.97; *P* < 0.00001; *I*^2^ = 90%). Sensitivity analyses were further conducted with Stata15.1 software to find the potential sources of heterogeneity. The results showed that one trial (Qiao, 2016) increased overall heterogeneity by 10 percent. In short, the MMSE of the HBOT group can be improved more effectively than that of the CT group as shown in Figure 4.

#### ADL Score: Addition of HBOT vs. Conventional Therapy

**Figure 5:**
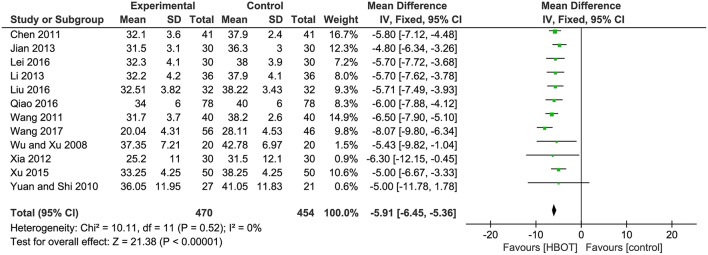
Comparison of the ADL score after treatment between the HBOT group and the control group.

There were 12 RCTs with a total of 924 (47.2%) patients recording the ADL score. Meta-analysis indicated that patients in the HBOT (*n* = 470, 47.8%) group benefit more than those in the conventional therapy group (*n* = 454, 46.8%) in terms of ADL score. (*MD* = −5.91; 95% *CI* = −6.45 to −5.36; *P* < 0.00001; heterogeneity: *P* = 0.52, χ^2^ = 10.11, *I*^2^ = 0%), as shown in Figure 5.

#### BADL Score: Addition of HBOT vs. Conventional Therapy

**Figure 6:**
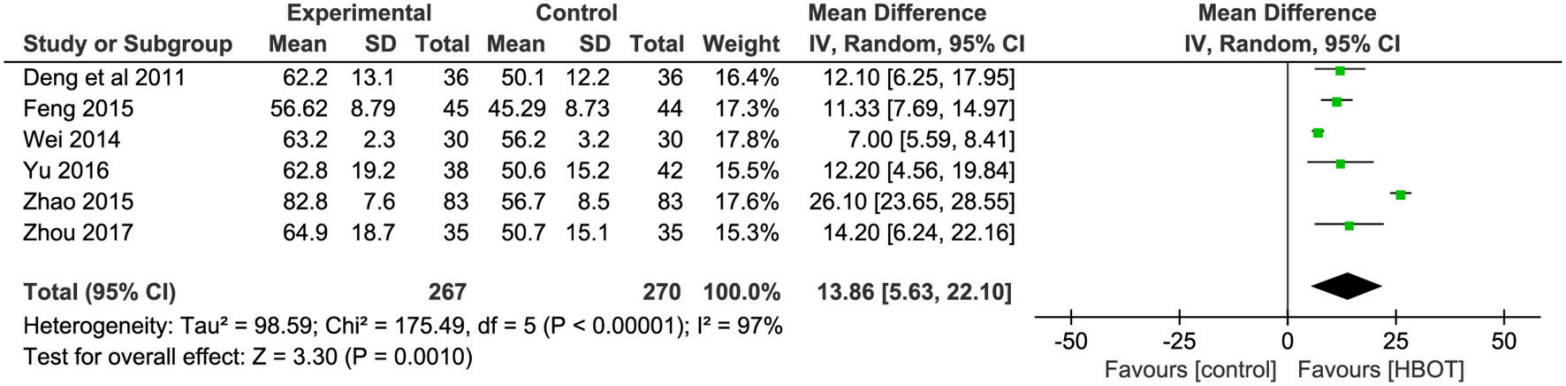
Comparison of the BADL score after treatment between the HBOT group and control groups.

The BADL was measured with the Barthel index method in six other trials, including Deng et al. (2011), Wei (2014), Feng (2015), Zhao (2015), Yu (2016), and Zhou (2017). The results showed the participants in the HBOT group (*n* = 267, 27.2%) had a statistically significant higher BADL score than patients in the conventional group (*n* = 270, 27.8%) but with substantial heterogeneity (*MD* = 13.86; 95% *CI* = 5.63–22.10; *P* = 0.001; heterogeneity: *P* < 0.00001, χ^2^ = 175.49, *I*^2^ = 97%). After sensitivity analyses by removing two articles (Wei, 2014; Zhao, 2015), no heterogeneity was observed and the outcomes of BDAL between the HBOT group (*n* = 154, 15.7%) and control group (*n* = 157, 15.9%) remained stable (*MD* = 11.93; 95% *CI* = 9.24–14.63; *P* < 0.00001; heterogeneity: *P* = 0.94, χ^2^ = 0.42, *I*^2^ = 0%), as shown in Figure 6.

### Secondary Outcomes

#### Hemorheology: Addition of HBOT vs. Conventional Therapy

**Figure 7:**
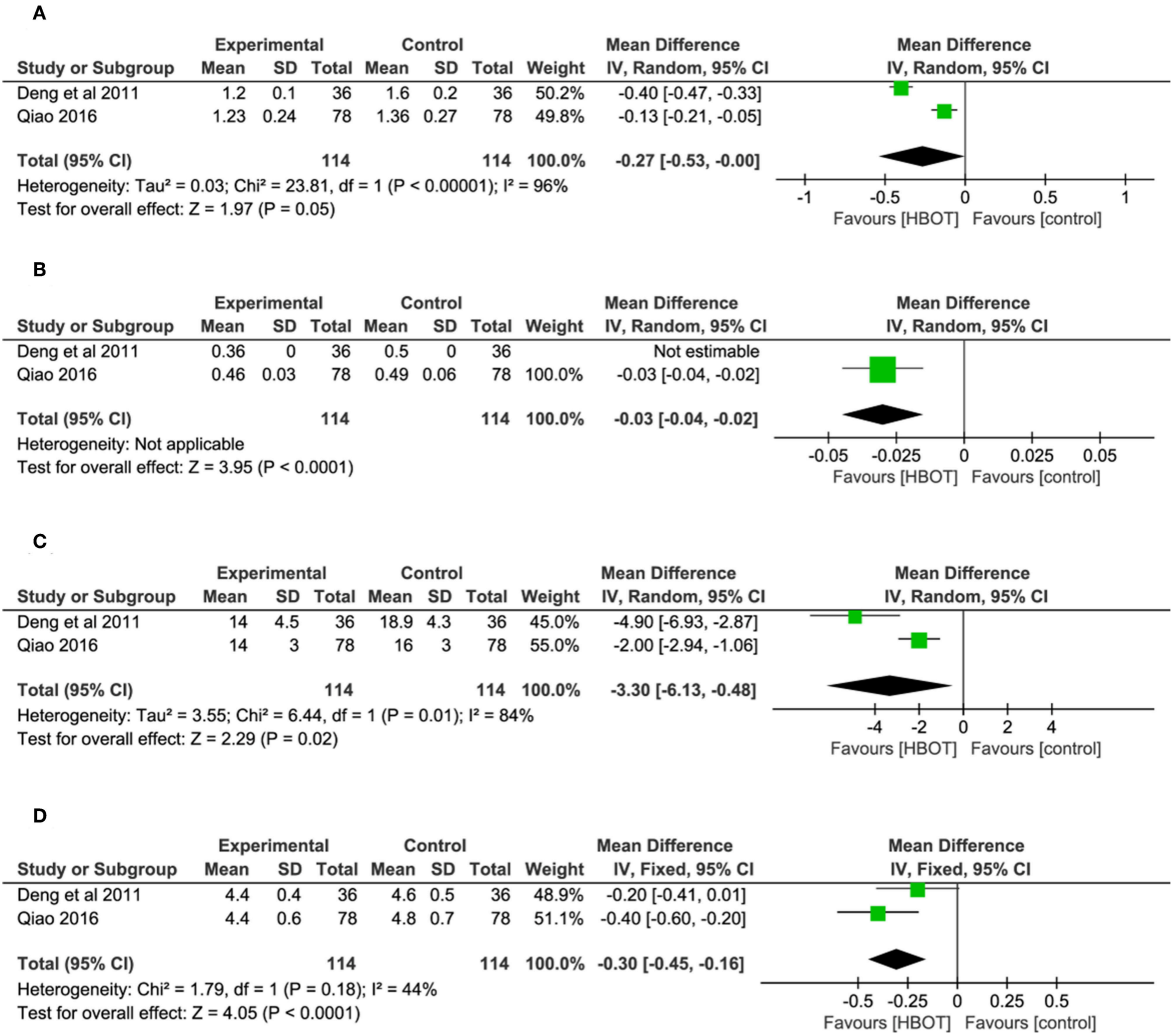
Comparison of the Hemorheology after treatment between the HBOT group and control groups. **(A)** Plasma viscosity, **(B)** hematocrit value, **(C)** erythrocyte sedimentation rate (ESR), and **(D)** fibrinogen.

The specific indicators of Hemorheology, including plasma viscosity, hematocrit value, erythrocyte sedimentation rate (ESR) and fibrinogen, were reported in two documents (Deng et al., 2011; Qiao, 2016), with 114 patients in each group. In comparison with the CT group, the HBOT group showed a greater reduction in the four indicators of Hemorheology, as shown in Figure 7 and Table 2.

**Table 2:** The meta-analysis result of hemorheology.

**Secondary outcome**	**95% Cl**	***P*-value**		**Study heterogeneity**		
			**χ^**2**^**	**df**	***I*^**2**^, %**	***P*-value**
Plasma viscosity	*MD* −0.27 [−0.53, −0.00]	0.05	23.18	1	96	<0.00001
Hematocrit value	*MD* −0.03 [−0.04, −0.02]	<0.0001	–	–	–	–
Erythrocyte sedimentation rate	*MD* −3.30 [−6.13, −0.48]	0.02	6.44	1	84	0.01
Fibrinogen	*MD* −0.30 [−0.45, −0.16]	<0.0001	1.79	1	44	0.18

#### Adverse Events

**Figure 8:**
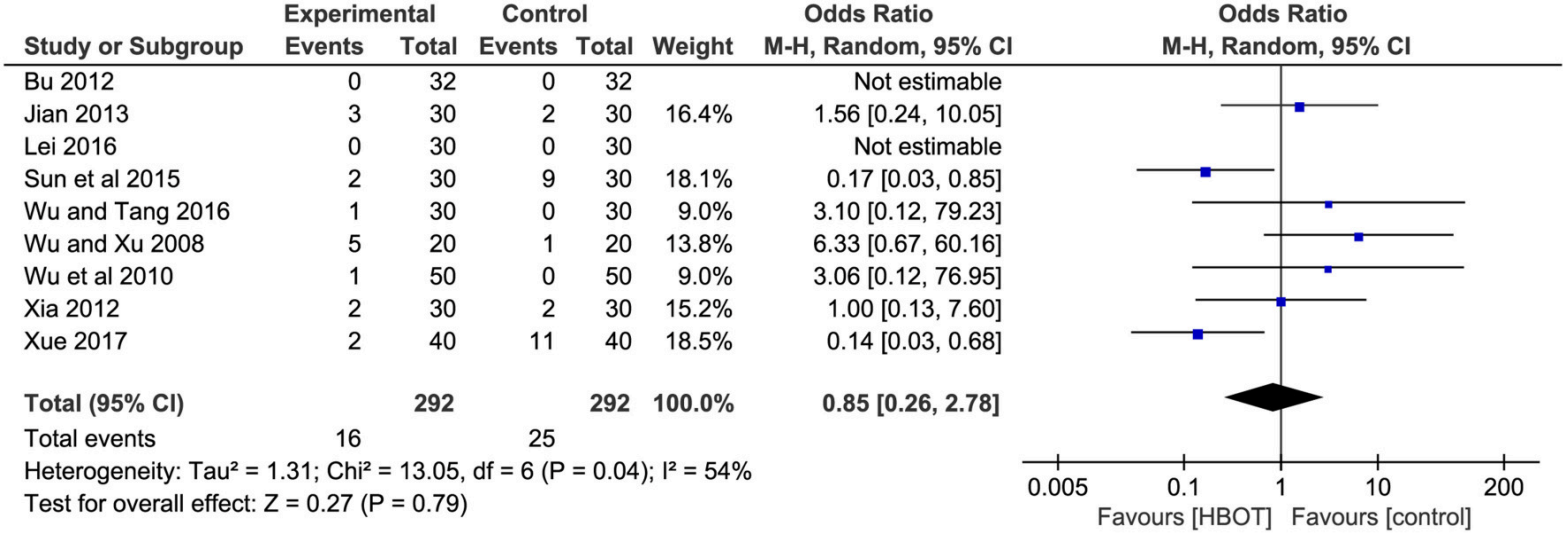
Comparison of the adverse events after treatment between the HBOT group and control groups.

Adverse events were reported in 12 trials (Wu and Xu, 2008; Jing and Luo, 2009; Wu et al., 2010; Yuan and Shi, 2010; Bu, 2012; Xia, 2012; Jian, 2013; Sun et al., 2015; Zhao, 2015; Lei, 2016; Wu and Tang, 2016; Xue, 2017). Three of them (Jing and Luo, 2009; Yuan and Shi, 2010; Zhao, 2015), with a total of 279 patients, recorded the specific adverse events, including nausea, vomiting, dizziness headache, insomnia, and elevated alanine transaminase (23/279, 11.5%), but they failed to identify which group the adverse reactions were associated with. Therefore, adverse events (HBOT: 16/292, 5.5%; CT: 25/292, 8.6%) with adequate information were detailed in nine studies, and two of them (Bu, 2012; Lei, 2016) declared there were no adverse events in their trial. The most frequent adverse reactions mentioned in these studies were gastrointestinal discomfort symptoms (nausea, diarrhea and abdominal pain) and dizziness, anxiety, insomnia and drowsiness. One trial (Wu et al., 2010) reported 1 case with elevated blood pressure, increased heart rate and rhinorrhagia in HBOT group. Another article (Wu and Tang, 2016) reported 1 patient with tinnitus and palpitations in the experimental group. In addition, patients experienced mental abnormalities in two studies (Sun et al., 2015; Xue, 2017). In general, all the adverse reactions were mild, and no serious adverse reactions were reported. The result of meta-analysis indicated no statistic difference between the two groups, suggesting that the addition of HBOT did not increase adverse events. (*OR* = 0.85, 95% *CI* = 0.26–2.78, *P* = 0.79), as shown in Figure 8.

#### Sensitivity Analysis

**Figure 9:**
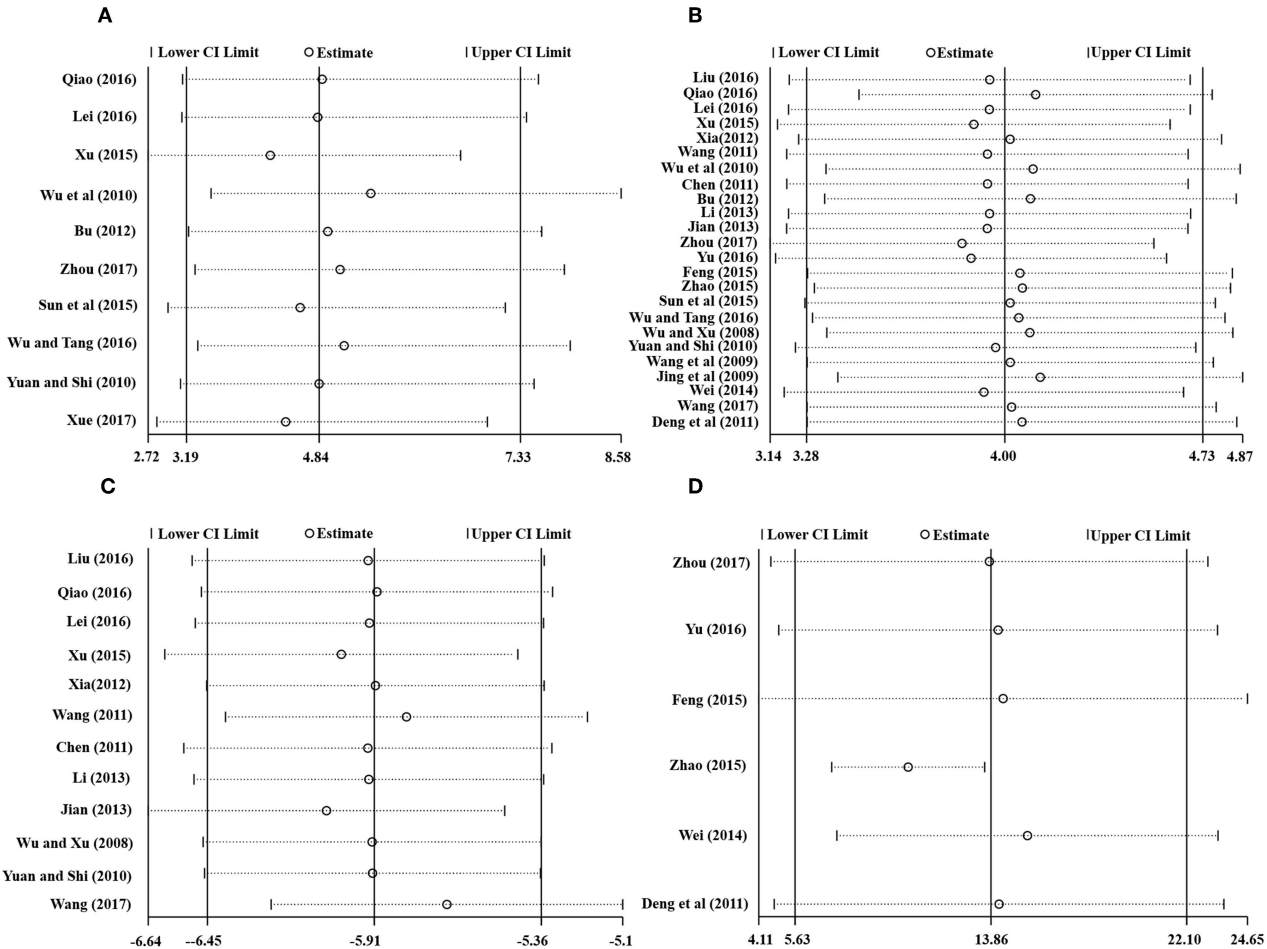
Sensitivity analysis plot of **(A)** TEF, **(B)** MMSE score, **(C)** ADL score, and **(D)** BADL score.

Stata 15.1 was employed for sensitivity analysis of the main outcomes, including TEF, MMSE score, ADL score, and BADL score. The results showed that removing any one study of each outcome had no significant effect on the overall results, indicating that the results of this meta-analysis were reliable, as shown in Figure 9.

#### Publication Bias

**Figure 10:**
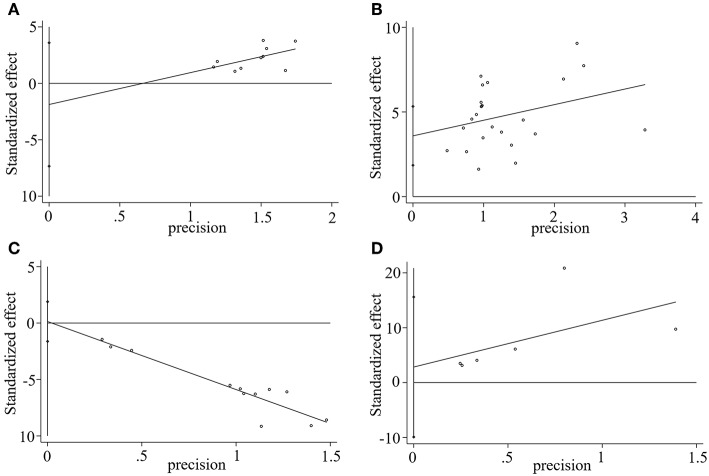
Egger's publication bias plot of **(A)** TEF, **(B)** MMSE score, **(C)** ADL score, and **(D)** BADL score.

**Figure 11:**
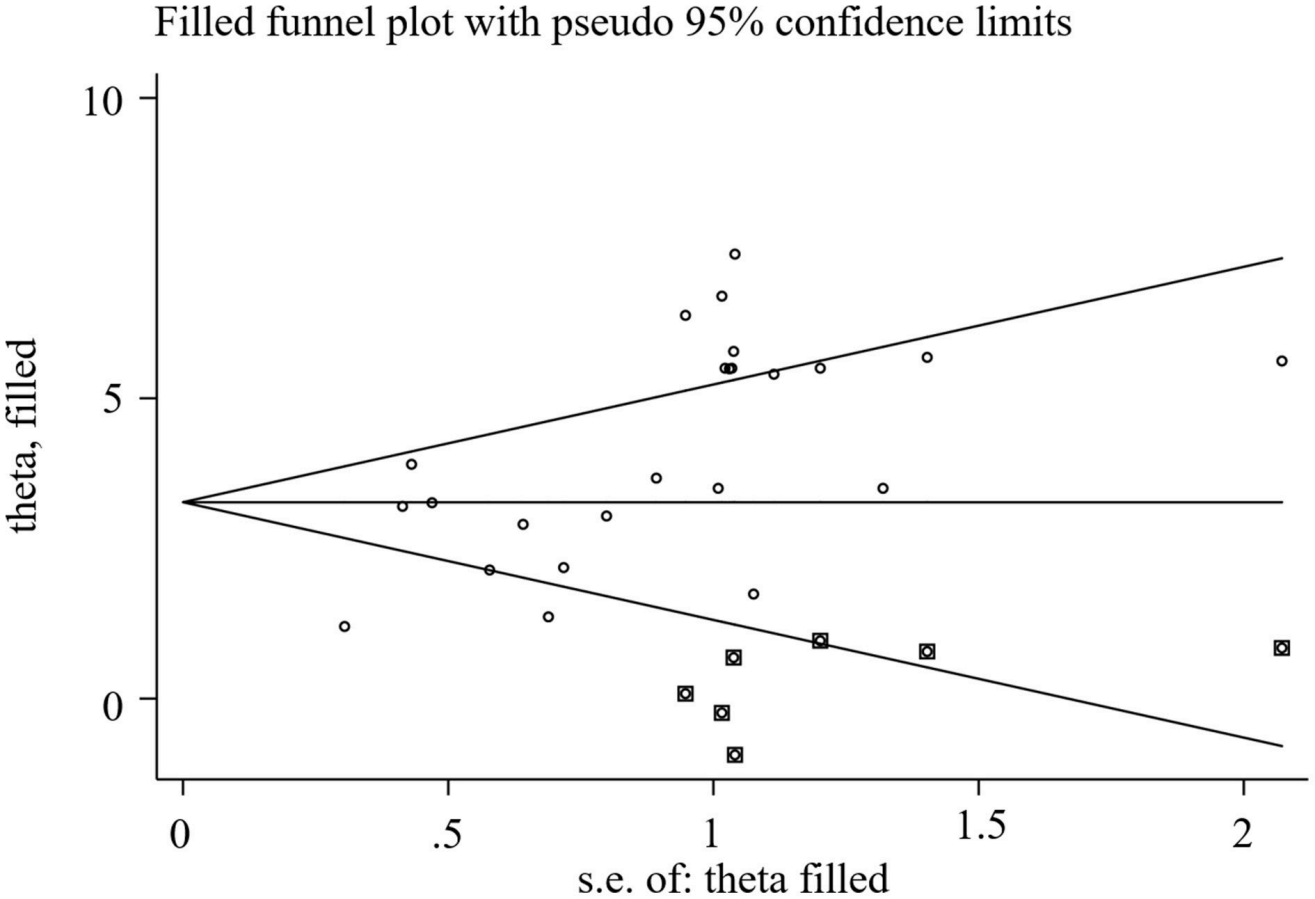
Filled funnel plot of MMSE score.

We used Stata15.1 software to detect the possible publication bias of primary outcomes, and trim and filling method was conducted to cope with striking publication bias if *P* < 0.05. The result of Egger's test suggested that there was no publication bias in terms of TEF (*P* > | *t* | = 0.452, 95% *CI* = −7.34 to 3.59), ADL score (*P* > | *t* | = 0.869, 95% *CI* = −1.62 to 1.89), BADL score (*P* > | *t* | = 0.571, 95% *CI* = −9.92 to 15.59) and Adverse events (*P* > | *t* | = 0.056, 95% *CI* = −0.16 to 8.36). For the MMSE score, significant publication bias was observed (*P* > | *t* | = 0.0001, 95% *CI* = 1.85–5.32) (Figure 10), and trim and filling method were used to evaluate the reliability of results affected by significant publication bias. After running the iterations, seven studies marked with squares in Figure 11 were filled. However, the *MD* and 95% *CI* after trim and filling method (*MD* = 3.27; 95% *CI* = 2.58–3.96; *P* < 0.00001) was consistent with the previous result (*MD* = 4.00; 95% *CI* = 3.28–4.73; *P* < 0.00001), indicating that the result was stable without flip.

## Discussion

VD is a complex syndrome with varied pathogenesis, including infarct dementia, microvascular ischemia disease, poor perfusion and hemorrhage, mixed dementia and cerebral autosomal dominance, with subcortical infarcts and leukoencephalopathy (CADASIL) (O'Brien, 2006). Commonly used diagnostic criteria include NINDS-AIREN, ICD10, DSM-IV (Chen and Zhang, 2016). Computed tomography and magnetic resonance imaging can, to some extent, indicate the progression of strategic infarct dementia, lacunar infarction and periventricular damage of white matter. Due to a lack of ideal biochemical indexes in the clinical diagnosis of VD, there were no unified clinical diagnostic criteria worldwide, and pathological examination was the only gold standard. Generally, demographic factors, genetic factors, general vascular risk factors and stroke-related factors play an important role leading to VD. A systematic review found that the first stroke of patients caused 10% VD, and at least 33.3% of VD resulted from palindromic stroke. However, current treatments focusing on reducing modifiable risk factors and neuroprotection are unsatisfactory. Therefore, finding clinically effective complementary therapies with lower adverse events can enhance the therapeutic efficacy of conventional therapies in treating VD.

HBOT, as an adjuvant treatment, has been widely used in cerebral injury and has shown great effects on reducing the disability rate and improving the cure rate. Because Hypoxia is one of the major pathological factors that leads to neuronal cell injury, HBOT achieves physiologic effects by increasing the oxygen level, raising oxygen tension, decreasing intracranial pressure and relieving brain edema. At the cellular level, animal studies suggested that HBOT decreased COX-2 mRNA and protein levels, and inhibited COX-2 overexpression in rats with cerebral ischemia (Yin et al., 2002). Additionally, the neuroprotection of HBOT is also associated with antioxidant effects and the reduction of apoptosis related to reducing the caspase-3 expression and activity (Yin et al., 2003; Calvert et al., 2004). A 2012 Cochrane system review was undertaken to address the efficacy and safety of HBOT for VD and concluded that the addition of HBOT for VD appeared to be more effective than the controls. However, the evidence obtained by them was probably unpersuasive because only one trial with 64 patients was analyzed in their review. Another systematic review (Chen and Zhang, 2016) conducted in China in 2016, demonstrated that applying HBOT as an adjunctive therapy strikingly improved the MMSE, ADL and HDS scores in VD patients. Despite the positive findings, their conclusion was not dependable because some studies included in their review were seriously flawed. For instance, there was a significant difference in the ADL score before intervention between the HBOT and CT groups in one of the included studies (Song, 2012), and the data from another study included in their review were obviously inconsistent (Bao and Zhong, 2007). Moreover, because only two of the 16 studies reported the adverse events, the conclusion regarding safety assessment of HBOT for VD was unconvincing. Therefore, we rigorously performed an updated systematic review to assess the effectiveness and safety of HBOT.

This updated meta-analysis assessed the evidence from 25 RCTs with a total of 1,954 VD patients randomized to receive additional HBOT or CT between 2008 and 2017. The main results included the following: (1) MMSE score were strikingly high in the HBOT group when compared with the CT group, (2) ADL score significantly favored the HBOT group compared with the CT group, (3) for the BADL, patients in the HBOT group benefited more than those in the CT group, (4) compared with patients treated with CT, the addition of HBOT resulted in a striking improvement in the TEF, (5) the HBOT group also showed additional benefits for improving hemorheology (plasma viscosity, hematocrit value, erythrocyte sedimentation rate and fibrinogen), and (6) the results across various subgroups were highly in agreement, and the benefits of HBOT were significant. No statistically significant difference was found between the HBOT and CT groups regarding the adverse events rate.

### Subgroup Analysis

To determine the best treatment strategy of HBOT, subgroup analysis of main outcomes was introduced according to the daily oxygen intake and treatment duration. The results showed a higher effect size value and narrower confidence interval of the “7–8 weeks” group than that of the “3–4 weeks” and “12–16 weeks” group, which indicated that “7–8 weeks” of treatment duration bring the maximum therapeutic effect to the VD patients, and the results are more precise and reliable, especially subgroup analysis of ADL. Subgroup analysis of daily oxygen intake suggested that there was a significant difference between the 60 and 120 min groups. Despite the significant difference favoring the 60 min group, it is unreasonable to conclude that 60 min of HBOT was more effective than 120 min because the number of studies between subgroups varied widely. However, considering the compliance and medical burden of patients and the fact that 60 min of oxygen inhalation was sufficiently effective, 60 min of daily HBOT with 7–8 weeks of treatment duration was recommended for the treatment of VD, as shown in Table 3.

**Table 3:** Subgroup analysis for oxygen intake and treatment duration.

**Subgroups**	**Trials**	**Effects model**	**Pooled effect**	**95% Cl**	***P-*value**
**TEF**					
Treatment duration (3–4weeks)	7	Fixed	OR 3.56	2.11, 6.00	<0.00001
Treatment duration (7–8weeks)	1	Fixed	OR 12.31	3.38, 44.89	0.0001
Treatment duration (12–16weeks)	2	Fixed	OR 6.77	2.91, 15.74	<0.00001
Total	10	Fixed	4.84	3.19, 7.33	<0.00001
Test for subgroup differences: Chi^2^ = 3.93. df = 2 (*P* = 0.14). *I*^2^ = 49.1%					
**MMSE SCORE**					
Oxygen intake (60 min, qd)	22	Random	MD 4.10	3.35, 4.85	<0.00001
Oxygen intake (120 min, qd)	2	Random	MD 3.04	−1.04, 7.12	0.14
Total	24	Random	MD 4.00	3.28, 4.73	<0.00001
Test for subgroup differences: Chi^2^ = 0.25. df = 1 (*P* = 0.62). *I*^2^ = 0%					
Treatment duration (3–4weeks)	10	Random	MD 3.42	2.28, 4.45	<0.00001
Treatment duration (7–8weeks)	7	Random	MD 5.53	4.73, 6.33	<0.00001
Treatment duration (12–6weeks)	7	Random	MD 3.37	2.65, 4.08	<0.00001
Total	24	Random	MD 4.00	3.28, 4.73	<0.00001
Test for subgroup differences: Chi^2^ = 17.64. df = 2 (*P* = 0.0001). *I*^2^ = 88.7%					
**ADL SCORE**					
Treatment duration (3–4weeks)	4	Fixed	MD −5.42	−6.43, −4.40	<0.00001
Treatment duration (7–8weeks)	6	Fixed	MD −6.13	−6.78, −5.48	<0.00001
Treatment duration (12–6weeks)	2	Fixed	MD −5.30	−8.99, −1.62	0.005
Total	12	Fixed	MD −5.91	−6.45. −5.36	<0.00001
Test for subgroup differences: Chi^2^ = 1.44. df = 2 (*P* = 0.49). *I*^2^ = 0%					
**ADVERSE EVENTS**					
Treatment duration (3–4weeks)	8	Random	OR 0.58	0.19, 1.77	0.34
Treatment duration (12–16weeks)	1	Random	OR 0.63	0.67, 60.16	0.11
Total	9	Random	OR 0.85	0.26, 2.78	0.79
Test for subgroup differences: Chi^2^ = 3.49. df = 1 (*P* = 0.06). *I*^2^ = 71.3%					

### Implications for Practice

Before we conducted this review, several studies had demonstrated the therapeutic effect of HBOT for treating brain-related diseases. An earlier animal experiment suggested that HBOT significantly improved learning, memory and recovery of blood perfusion in rats with VD by raising neurogenesis and cerebral blood flow in piriform cortex (Zhang et al., 2010). Then, a meta-analysis (Cui et al., 2017) relating to an intracerebral hemorrhage (ICH) animal model indicated that the HBOT group significantly reduced the brain water content (BWC) and improved the neuro-behavioral outcome (NO). In addition, another review including 51 trials for the animal experiment also showed that HBOT significantly improved neurological function and reduced the infarct size by 32% when compared with the CT group, and the mortality was 8.3% lower than that of the CT group (Xu et al., 2016). Almost at the same time, (Wang et al., 2016) reported that HBOT significantly improved the Glasgow coma scale (*MD* = 3.13, 95% CI 2.34–3.92, *P* < 0.001), Glasgow outcome score of patients with traumatic brain injury (*OR* = 3.78, 95 %*CI* 1.23–11.63, *P* = 0.020), and the overall mortality of HBOT group was strikingly lower than the CT group (*OR* = 0.32, 95 %*CI* 0.18–0.57, *P* < 0.001). Compared with the previous two reviews, this meta-analysis first reported the effects of HBOT on hemorheology, which further explained the neuroprotective mechanism of HBOT. However, only two studies reported the hemorheology data, and most studies lacked data on degenerative cellular and molecular parameters such as apoptosis, cell death, inflammation and amyloid accumulation. Therefore, the mechanism of HBOT warrants further investigation. On the whole, the available evidence obtained from our review showed that application of HBOT as adjuvant therapy has additional benefits on VD patients and is generally safe. Furthermore, our findings are consistent with previous reports, which provides important clinical evidence for clinicians.

### Limitations

Several limitations should be highlighted in our meta-analysis. First, we only searched the main English and Chinese databases. Therefore, some studies meeting our inclusion criteria published in other languages or databases may have been excluded. All included trials declared randomization, but only three studies described a specific randomization method. Blinded assessments were not detailed in all included documents, which may have exerted a potential impact on the objectivity of VD outcomes. Second, the inclusion criteria of these studies had small sample sizes with low-quality designs, which may give rise to overvaluing the benefit of HBOT. Additionally, there may be a certain degree of selective reporting bias because most studies had not been officially registered. Third, only two eligible studies reported the hemorheology. Fourth, 13 papers did not mention any information on adverse reactions. Thus, the safety assessment of additional HBOT in treating VD was unsatisfactory. Fifth, although the simple mental state examination scale (MMSE), first introduced by Folstein in 1975, has been widely used as a screening tool for dementia and mental disorders in hospitalized patients, the evaluation of therapeutic effect using MMSE was restricted because cross-cultural translation of MMSE is not reliable, and the translation is likely to be confusing due to language, script skills, culture and ethical norms, especially for patients with <5 years of education. However, although the deficiency listed above may undermine the quality of evidence, the included trials are highly comparable, and the documents were selected in relatively strict inclusion criteria. Since the patients of selected studies were mainly from China, the conclusion of this meta-analysis is not applicable to other ethnic groups. Therefore, large sample trials with high-quality and well-designed ethnic groups should be conducted in the future to provide more reliable evidence regarding the efficacy and safety of HBOT for VD.

## Conclusion

The present evidence from this meta-analysis suggested that the addition of HBOT to standard conventional therapies for VD significantly improved the MMSE, ADL, BADL, hemorheology, and clinical efficacy. In view of the effectiveness and safety of HBOT, it is reasonable to recommend HBOT as a complementary therapy for the treatment of VD.

## Author Contributions

QY conceived this review and completed the manuscript. LL performed the literature searches electronically and manually. NY and DL performed study selection and data extraction. YY and SX assessed the risk of bias. YL and HC critically revised the paper.

### Conflict of Interest Statement

The authors declare that the research was conducted in the absence of any commercial or financial relationships that could be construed as a potential conflict of interest.
